# Does Caregivers' Responsiveness Influence the Early Development of Deictic Gestures? Longitudinal and Training Evidence in Relation to Subsequent Language Development

**DOI:** 10.1111/infa.70112

**Published:** 2026-08-17

**Authors:** Katharina Kaletsch, Ulf Liszkowski

**Affiliations:** ^1^ Department of Developmental Psychology University of Hamburg Hamburg Germany

**Keywords:** communicative development, infant gestures, pointing, responsiveness, training

## Abstract

Infants' deictic gestures, like hold‐out‐give (HoG) and pointing, are early manifestations of intentional, referential communication and predict later language acquisition. But what factors predict the early emergence of these deictic gestures? The present study investigated the longitudinal and cross‐sectional influence of caregivers' responsiveness and pointing, beginning at 7 months, on infants' HoG and index‐finger pointing gestures at 10 months, and infants' language abilities at 24 months. Utilizing a longitudinal design, we assessed 82 typically developing *German* infants, 42 of whom were randomly assigned to an intervention group targeted to increase caregivers' responsiveness to their infants' communicative signals and object‐directed interactions. We hypothesized that in a process of social co‐construction caregivers' responsiveness would enhance infants' gesture and language development. In addition, we examined the hypothesis that infants learn about reference by observing caregivers' initiative pointing gestures, in which case we expected positive associations between caregivers' initiative pointing frequency and infants' communicative development. Group comparisons at 10 months revealed increased HoG frequency in the responsiveness training group. Pointing frequency or subsequent language skills at 24 months did not differ between groups. Regression analyses revealed a positive association between caregivers' responsiveness at 10 months and infants' HoG frequency across groups. Analyses revealed no association between caregivers' initiative pointing and infants' communicative development. A predictive relation between infants' pointing frequency and their subsequent language abilities was confirmed. In support of social constructivist positions, these findings indicate that already infants' early deictic gesture use, specifically their HoGs, is influenced by caregivers' responsiveness.

## 1 Introduction

In the latter half of the first year of life, infants begin to use deictic gestures to direct the attention of other persons toward external entities. At approximately 10 months of age, infants begin to hold out and give objects to their interaction partners (HoG gestures) in an effort to share interest and proactively initiate episodes of joint attention (Boundy et al. 2019; Cameron‐Faulkner et al. 2015, 2020). At approximately 11–12 months of age, infants expand their communicative repertoire to refer to distant and even absent entities via index‐finger pointing (Liszkowski et al. 2007; Liszkowski et al. 2009; Rüther and Liszkowski 2023). Although HoG and pointing gestures differ in their level of complexity regarding their immediate versus deferred connection to a referent object, they represent early manifestations of intentional object‐related communication. Both of these deictic gesture types have been shown to predict subsequent language acquisition (Cameron‐Faulkner et al. 2020; Choi and Rowe 2021; Lüke et al. 2017). However, still little is known about the predictors of interindividual variance in the early emergence of infants' object‐related gestures (Rüther and Liszkowski 2023; Salter and Carpenter 2022).

One proposal posits that infants learn to communicate in more complex and frequent ways through active social co‐construction with their caregivers (Bruner 1983; Lock et al. 1990). Within this social constructivist framework, different caregiver interaction styles have been discussed to enhance infants' communicative development (for a review, see Liszkowski and Rüther 2021). On the one hand, caregivers' contingent and relevant responses to infants' communicative signals may constitute feedback processes of learning which enhance infants' subsequent communicative development and language acquisition (Alvarenga et al. 2021; Elmlinger et al. 2023; Luchkina and Xu 2022; Tamis‐LeMonda et al. 2014). On the other hand, caregivers' attention‐directing pointing gestures (i.e., initiative points, as opposed to responsive points) may serve as a model from which infants learn about the attention‐directing, referential function of gestures through processes of emulation and imitation (Clark and Kelly 2022; Kee 2020; Rowe and Leech 2018). Further, some researchers suggest that infant gestures are socialized from an initial, non‐communicative usage into a final, communicative usage– they are shaped through responses into something new (e.g., Carpendale and Carpendale 2010)—but only once these gestures have already emerged, which leaves open how these gestures would first emerge. It is currently unknown whether a process of social co‐construction runs deep in ontogeny and applies even to the early, first emergence of object‐related gestures, and whether the process relates to caregivers' responsiveness or their initiative attention‐directing gestures.

## 2 Effects of Caregivers' Responsiveness

Several findings support the notion that social co‐construction processes drive early communicative development, especially regarding caregivers' responsiveness (Alvarenga et al., 2021; Elmlinger et al., 2023). With respect to infants' HoG gestures, research has indicated that 10‐month‐old infants adapt their communicative behaviors based on whether or not their previous HoG gestures were responded to by joint attention (Boundy et al. 2019). An intervention study targeting caregivers' responsiveness in 6‐month‐old infants resulted in more infant prelinguistic communication, including HoG and pointing gestures, at 12 months compared to an active control group (Salter et al. 2023). However, HoG gestures emerge around 2 months earlier, and so it remains less decisive whether caregivers' responsiveness enhances infants' deictic gesture development from its very beginning.

With respect to infants' pointing gestures, cross‐sectional experimental studies have found that 12‐month‐olds directly adapt their pointing frequency to the responses elicited by their previous pointing (Kaletsch and Liszkowski 2024b; Liszkowski et al. 2004; Miller and Lossia 2013; Wu and Gros‐Louis 2014). A longitudinal study demonstrated that caregivers' responses that addressed the targets of infants' gestures at 10 months predicted infants' pointing at 12 months (Ger et al. 2018). Further, caregivers' longer interaction responses to infants' HoG gestures at 10 and 11 months were longitudinally related to more index‐finger pointing in infants 1 month later (Cameron‐Faulkner et al. 2015). An intervention which aimed at enhancing caregivers' responsiveness to infant pointing at 12 months lead to an increase in infants' pointing frequency 1 month later (Kaletsch and Liszkowski 2025). These findings thus show that caregivers' responsiveness influences infants' pointing frequency from the first birthdays on. It remains to be investigated whether caregivers' responsiveness to earlier forms of infant communication predicts, and may even be used to enhance, pointing development before 12 months of age.

With respect to language, the promoting effect of caregivers' responsiveness is relatively uncontested and has been established with regard to various age groups, paradigms, and samples (Bornstein et al. 2020; Hudson et al. 2014; Levickis et al. 2023; Smith et al. 2022). However, it remains to be investigated whether caregivers' early responsiveness continues to explain variance in infants' later language development when controlling for the predictive value of infants' preceding pointing (Cameron‐Faulkner et al. 2020; Colonnesi et al. 2010; Lüke et al. 2017) and HoG frequency (Choi and Rowe 2021).

## 3 Effects of Caregivers' Pointing

Findings on the role of caregivers' pointing as a model for acquiring referential communication, or specifically the pointing gesture, are less decisive. With respect to infants' HoG development, Salomo and Liszkowski (2013) found that infants' HoG frequency differed across cultural groups (Mayan, Dutch and Chinese) as a function of interactional input, including—but not restricted to—caregiver pointing. At 14 months, infants' general gesture use (including HoGs) during home visits was found to correlate with caregivers' gesture types (Rowe and Goldin‐Meadow 2009; Rüther and Liszkowski 2023 found no significant correlation between caregivers' pointing for their 8‐month‐old infants in the decorated room and the infants' subsequent HoG use. Further empirical research is needed to examine the specific influence of caregivers' initiative pointing on infants' HoG development.

With respect to infants' pointing development, caregivers begin to point for their infants around 7 months of age (Rüther and Liszkowski 2023). Cross‐sectional studies have reported on positive correlations between caregivers' and infants' pointing around 12 months during free play (Matthews et al. 2012; Rowe and Leech 2018) and in the decorated room (Liszkowski et al. 2012; Liszkowski and Tomasello 2011; Rüther and Liszkowski 2023). These cross‐sectional relations are not consistently found across all studies and settings (Salo et al. 2019; Rowe 2000; Ger et al. 2018; Kaletsch and Liszkowski 2024a, 2024b, 2025 [using an online remote variant of the decorated room]). Longitudinally, Rüther and Liszkowski 2023 found that parental pointing at 8 months in the decorated room correlated with infants' pointing onset. Another longitudinal study did not confirm the predictive value of caregivers' pointing in the decorated room on infants' pointing development (Ger et al. 2018, 2023, same sample). Rowe and Leech (2018) found that training caregivers to increase their pointing at 10 months enhanced caregivers' and infants' pointing at 12 months. A comparable training study found no enhancing effect of increased caregiver pointing on infants' pointing (Matthews et al. 2012). These somewhat mixed results are likely influenced by the contexts of data collection, coding, analyses, and sample sizes. Further, caregivers' pointing is often not differentiated into responsive gestures (contingently following into infant's established focus) and initiative gestures (initiating a new focus), which makes it difficult to distinguish a purely modeling function from effects of responsiveness. While the majority of caregiver pointing is typically initiative (Liszkowski et al. 2012; Kishimoto 2017), the specific relationship between caregivers' initiative pointing and infant pointing remains to be further investigated, especially with regard to the early development of infant gestures prior to 12 months of age.

With respect to infants' language development, de Villiers Rader and Zukow‐Goldring (2012) found that caregivers' HoG gestures increased infants' successful mapping between words and referents. Longitudinally, caregivers' gesture use at 12 months was found to predict infants' language abilities at 18 months (Talbott et al., 2015). Specifically, caregivers' pointing frequency at 12 months predicted child vocabulary at 18 months (Choi and Rowe, 2021), and this relationship remained stable when assessing children's vocabulary at 36 months (Choi et al. 2021). Rowe and Goldin‐Meadow (2009) reported a relation between caregivers' and infants' gestures, which in turn predicted infants' language development. Further research is needed to establish whether caregivers' early pointing directly influences infants' language development.

## 4 The Current Study

Most previous studies on the relation between infants' communicative development and caregivers' behavior have faced certain limitations. Firstly, by investigating either caregivers' responsiveness or caregivers' pointing, the conceptual overlap is often not considered. Responsiveness includes the gestural modality, and while pointing gestures may often be used to initiate attention‐directing communication, they can certainly also be used in a responsive way, contingently on infants' focus of communication and activity. A recent study suggests that approximately 40% of caregivers' responses include gestures (van der Klis et al. 2023). Secondly, by relying on a single paradigm, findings may be affected by paradigm specific biases (e.g., more responsive behavior during free play vs. more directing behavior in contexts like book‐reading or the decorated room). Thirdly, regarding caregiver‐based training studies, it is necessary to clearly distinguish the direct influence of caregivers' changed behaviors on infants' gesture frequency during the outcome assessment, that is cross‐sectionally, from long‐term effects stemming from the longitudinal experiences of caregivers' changed behaviors over time (Boundy et al. 2019; Kaletsch and Liszkowski, 2024b; Miller and Lossia, 2013).

The current study investigated the role of caregivers' responsiveness and their initiative pointing in the early development of communication in the context of a longitudinal study including an intervention group, in which parents were educated about the potential benefits of responsiveness and explicitly asked to be responsive to their infants in their daily interactions. Caregivers' responsiveness was conceptualized as a contingent change of caregivers' behaviors across modalities (e.g., gestures, facial expressions, speech) as a response to infant communication (gestures and vocalizations), that is infants' signals, incipient gestures and object‐directed interactions (Salter and Carpenter, 2022). Caregivers' initiative pointing was coded reversely as non‐contingent. We collected longitudinal data from infants' seventh to their 10th months of age. To observe a variety of infant and caregiver gestures we used remote variants of the decorated room (Kaletsch and Liszkowski, 2024a), inducive of pointing, and the free play setting, inducive of proximal object manipulation and HoG gestures. Remote testing enabled us to acquire a sufficient sample size, minimized hurdles to participate in repeated lab visits, and increased convenience and naturalness through assessments in the home environment. After baseline data collection at 7 months, the training group was instructed through educational intervention with examples to increase their responsiveness to infants' signals and object‐directed interactions and incipient gestures, and encouraged to be responsive daily in at least a 15‐min interaction with their infants. The other group received no specific instructions in order to assess their typical, uninstructed natural development. Following a 3‐month period, we compared 10‐month‐old infants' HoG and early pointing frequency, as well as caregivers' behaviors, between groups. Additionally, we assessed during a follow‐up whether infants' language development at 24 months differed between groups.

If caregivers' responsiveness is a key component of a social co‐construction process already early in development, we expected positive longitudinal and cross‐sectional relations between caregivers' responsiveness and infants' HoG and pointing gestures at 10 months, and infants' language skills at 24 months. Further, we anticipated a promoting effect of enhanced responsivity in the training group on infants' HoG gestures, pointing, and language development. If caregivers' modeling of referential communication, beyond responsiveness, is a key component of acquiring referential communication, we anticipated positive relations between caregivers' initiative pointing and infants' HoG gestures, pointing, and language development. Further, we expected to reproduce previously reported positive relations between infants' gesture development and their subsequent language abilities (Colonnesi et al. 2010; Kirk et al. 2022).

## 5 Method

### 5.1 Participants

Participants were recruited from a database of families who had agreed to participate in developmental studies. Families typically range narrowly from middle to high socioeconomic backgrounds. The families lived in a *German* metropolis. All infants were typically developing, without visual or auditory impairment, and born at full term. We planned to collect data of 80 dyads to compare infants' communicative development between groups using independent *t*‐tests (*d* = 0.65; *α* = 0.05; Power = 0.8; calculated with G*Power). To account for dropouts, we invited 90 infant‐caregiver dyads. Eighty‐eight dyads participated at t1, of which five were excluded from analyses because the dyads did not attend subsequent appointments. One additional infant was diagnosed with visual impairment during the study and was therefore not included in the analyses.

The final sample included 82 participants (41 female infants). Forty dyads (21 female infants) were randomly assigned to the control group. At t1 infants' mean age was 231.4 days (SD = 4.3), and at t2 infants' mean age was 314.1 days old (SD = 10.1). Nine percent of all infants were raised bi‐ or multilingual, 31% of the infants had an older sibling, 4% of the infants visited daycare on a regular basis, and 6% of the caregivers were single caregivers. The mean age of the caregivers was 35 years (SD = 3.5). Socioeconomic status (SES) was assessed using the highest parental educational attainment (85% had at least a bachelor's degree), indicating that the sample was broadly representative of the middle to high socioeconomic range. Participants did not differ between groups in terms of infants' gender, age, or socioeconomic background. The present study was conducted according to guidelines laid down in the Declaration of Helsinki, with informed consent obtained from a caregiver for each child before any assessment or data collection. All procedures in this study were approved by the local ethics committee at the *University of Hamburg*.

### 5.2 Procedure

**FIGURE 1:**
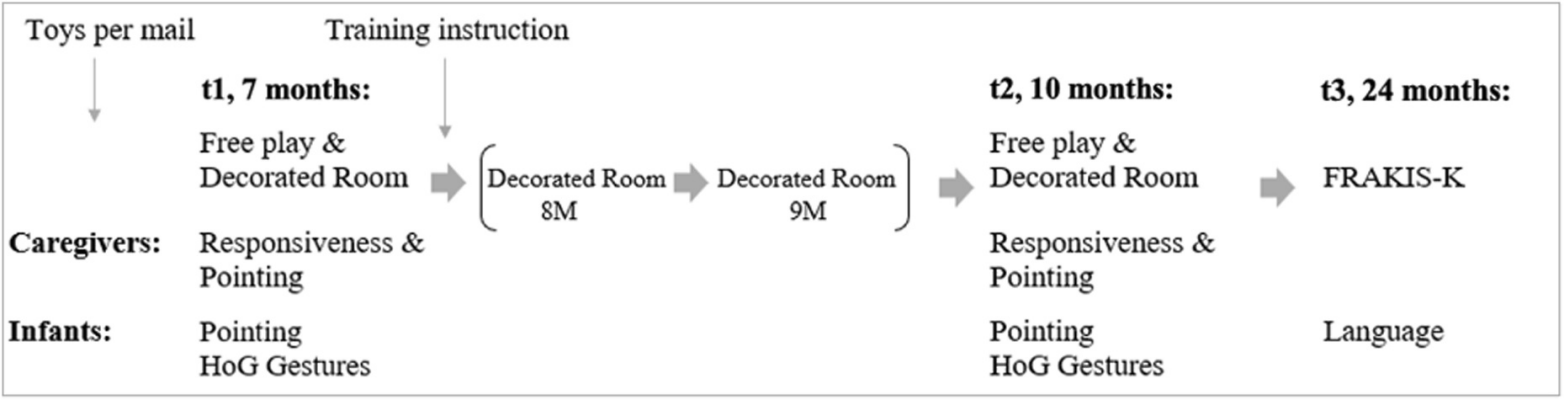
Study design. Data at t1 and t2 were collected during remote appointments in online video chat sessions. Language data at t3 was collected with an online survey.

We collected data at in online video chat sessions at infants' age of 7 months (t1) and infants' age of 10 months (t2). At infants' age of 24 months (t3), caregivers reported on their infants' language development via an online survey of the short version of the Questionnaire on Early Language Development (FRAKIS‐K, Szagun et al. 2023). As displayed in brackets in Figure 1, both groups participated in two additional remote decorated room appointments over the course of the study. These aimed at keeping the caregivers' involved in the training and offering regular opportunities to clarify questions. Prior to the first session, caregivers received an email with documents about the privacy policy, technical requirements, general instructions, and a link to a questionnaire about their socioeconomic status. Further, caregivers received toys for the free play session by mail. All caregivers provided informed consent to participate in the study. We invited the infants' primary caregiver (the person who spends the most time with the infant during the study period) to participate in data collection and training implementation. The study was conducted in accordance with the Declaration of Helsinki and was approved by the local ethics committee of the authors' institution.

**FIGURE 2:**
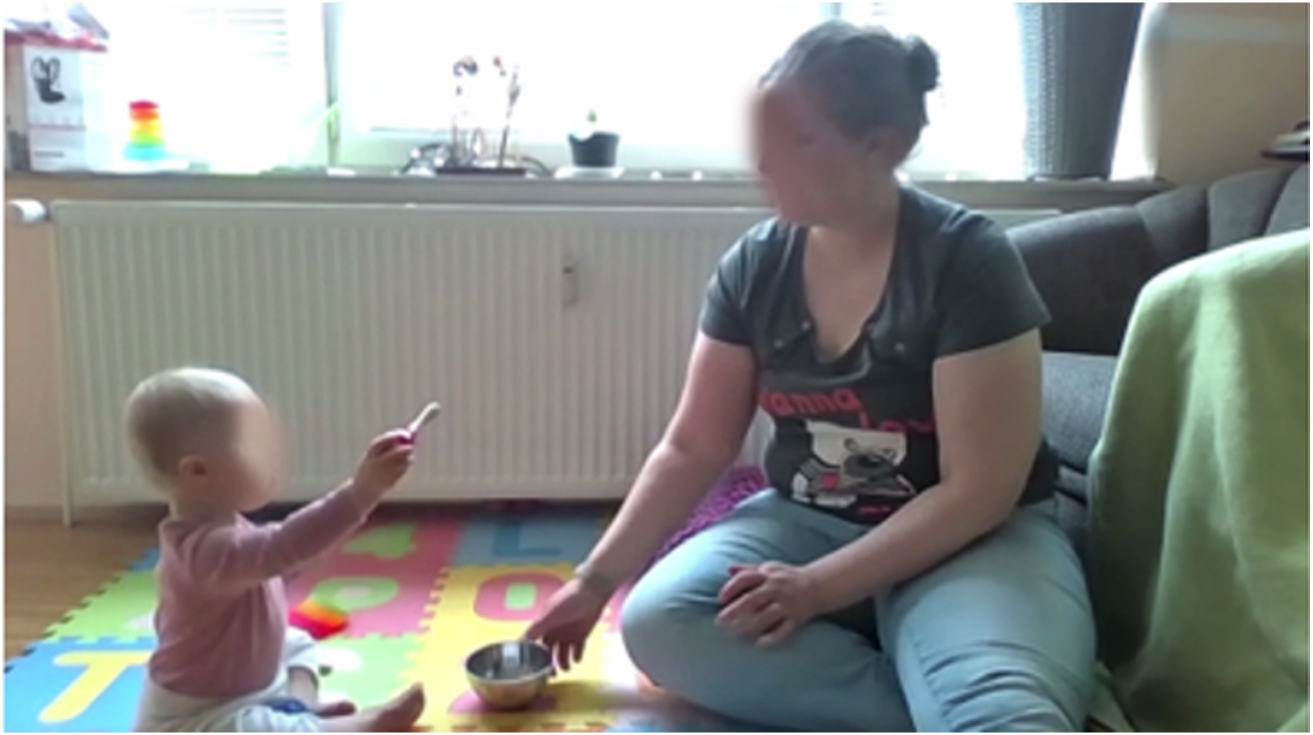
Infant's HoG gesture during remote free play at the 10‐month‐assessment.

During the video chat sessions, we first optimized the data collection conditions with regard to visibility and avoidance of distraction whenever necessary. We used five‐minute remote free play sessions to observe infants' HoG gestures. We selected toys that would encourage different types of play and be of interest to both 7‐ and 10‐month‐old infants. The set included four different colored wooden rings, a silver bowl, a wooden treasure chest, a porcupine ball, a rainbow spiral, a rattle, a spoon and a magic wand. Typically, caregivers placed the laptop on a chair or the sofa to transmit the scene from an elevated angle without distracting the infant (see Figure 2). Caregivers were instructed to “play with your infant as if no one is watching you”. Free play was conducted after the remote decorated room, with the order of the paradigms being reversed if the infants' mood required it.

**FIGURE 3:**
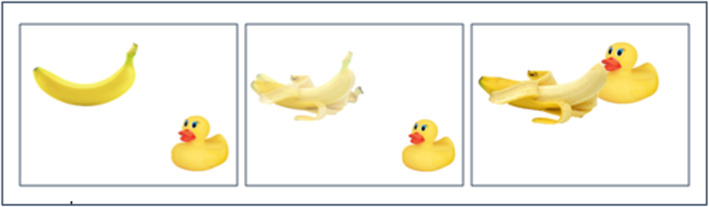
Sample stimuli in the remote decorated room. In the first animation sequence, the banana is peeled. In the second animation sequence, the rubber duck moves toward the banana

We used the remote decorated room paradigm to elicit infants' pointing (Kaletsch and Liszkowski, 2024a). Dyads watched a slide show together via screen sharing for 5 minutes while being webcam‐recorded at an appropriate distance to capture all gestures. Each slide showed two infant‐appropriate stimuli revealed by a page‐turning animation. After seven and 18 seconds, short animations, such as changes in color or position (see Figure 3), occurred on each slide. We implemented these animations to allow the dyads to discover something new during the presentation of each slide, thus increasing the likelihood of triadic communication. For the same reason, a face was shown on each slide. Four videos and five additional sounds were evenly distributed throughout the presentation. The presentation time of each slide was adapted to the dyads engagement and ranged from five to 30 seconds. The infants were either seated in a high chair next to their caregiver or on the caregivers' lap. Caregivers were instructed to “look at the following slides together with your infant and behave as if no one is watching you.”

We used the short version of the Questionnaire on Early Language Development (FRAKIS‐K, Szagun et al. 2023) to assess infants' language development. Caregivers completed an online survey when the infants reached 24 months of age. The survey contained 102 words (e.g., mouse, give, slow, how). The raw count of infants' expressive lexicon was transformed into gender and age‐specific percentiles with the corresponding table in the questionnaire's manual.

#### 5.2.1 Training

As shown in Figure 1, caregivers in the intervention group received training immediately after baseline data collection. The training instructions were delivered via a pre‐recorded eight‐minute video for comparability purposes. The video provided information on infants' gestural development and its relevance to language development. Further, we emphasized the influence of caregiver behavior on infants' communicative development and asked them to implement our training for at least 15‐min per day. The training involved identifying and/or establishing situations of shared interest in everyday life (e.g., morning routines, walks together). The training instructions were kept general to avoid enhancing a specific behavior on expense of another, and to keep the general flow of interaction as natural as possible. We asked caregivers to respond to infants' communication by i) sharing infants' focus of attention, ii) communicating about it, iii) temporal immediacy. After explaining the key components of the training, the instruction video showed an exemplary triadic interaction sequence. The instruction video ended with a brief recap. To assess caregivers' comprehension of the training, at the end of the instruction video we asked them i) how their infants communicate their interests, and ii) how the caregiver should respond according to the current training program. Any uncertainties were clarified and caregivers were emailed a link to return to the video at their convenience. The same email included the digital version of a protocol booklet that contained a weekly survey on caregivers' training frequency. On a scale of one to five, caregivers self‐reported that they practiced the training sometimes (3) to often (4) per week (*N* = 20, *M* = 3.65, SD = 0.59). A flyer and a detailed booklet, both with written training instructions, accompanied the mailed protocol booklet for the training group. The training group further received an additional email between two appointments containing information on infants' development, training suggestions, and a reminder of the protocol booklet to keep caregivers involved in the study. The training materials are available in *German* and English here [https://osf.io/sx84w/files/osfstorage].

We aimed at investigating whether increased caregivers' responsiveness promotes infants' communicative development in comparison to infants' natural interaction experiences. Therefore, caregivers in the control group received no specific instructions, allowing to control for potential general influences of longitudinal observations, like sample composition, repeated study participation, parental involvement, and potential others sources of noise, which were kept similar across the two groups which only differed in the instruction to parents.

#### 5.2.2 Coding and Reliability

Data were coded using Mangold Interact Lab Suite version 20.11.20.0. We coded the duration of each paradigm (remote decorated room and free play), infant fussing, and off‐task activities to maintain the duration of undisturbed data collection. We coded for infant HoG gestures when the infant held an object in the direction of the caregiver, regardless of any subsequent transfer of the object (give gesture, see Cameron‐Faulkner et al. 2015 & 2020). We coded infant index‐finger pointing when the arm was extended while the index‐finger was clearly extended from the other curled fingers (Liszkowski and Tomasello, 2011). Caregivers' behavior was coded as responsive when caregivers contingently changed their behavior (e.g., gestures, facial expressions, speech) in relevant ways in response to infant communication (gestures and vocalizations) within 2 seconds (Ger et al. 2018; Nicoladis and Barbosa, 2024; Wu and Gros‐Louis, 2014). To this end, we also coded infants' hand points, reaching gestures, and intentional vocalizations, as these were behaviors which we could reliably detect in the remote videos and to which caregivers could respond. Non‐responsive caregiver behaviors were not coded. They occurred when the caregiver ignored the infants' communicative signals, responded with a delay of more than 2 seconds, or redirected infants' attention away from their current focus.

Caregivers' initiative pointing was coded whenever the gesture did not occur within the 2 seconds after an infant signal (gesture or vocalization). Handshape was not further classified for caregivers' pointing gestures.

Nine coders were trained on the coding schemes. To assess inter‐rater reliability, we calculated Cohen's kappa for 20% of the videos (33 videos for each paradigm) using a tool integrated in the Interact software. We defined matching codes as two identical codes separated by a maximum of 2 seconds. Kappas were excellent for each coder for infant HoG gestures (*κ* > 0.81), infant index‐finger pointing (*κ* > 0.83), infants' other gestures and vocalization (*κ* > 0.78), caregivers' responsiveness (*κ* > 0.82), and caregivers' initiative pointing (*κ* > 0.85).

### 5.3 Analyses

Data were analyzed using RStudio (packages: betareg, MASS, haven, sandwich, lmtest, pscl, ggplot2). Infants' HoG frequency was calculated per minute of undisturbed data collection during free play to account for differences in the individual durations of recordings. Infants' pointing frequency was calculated per minute of undisturbed data collection in the remote decorated room. We refrained from combining infants' behavior across paradigms as the decorated room does not allow for HoG gestures by design (instruction not to touch the screen or distracting toys) and infants barely use pointing gestures during free play (Wu and Gros‐Louis 2014). Infants' caregiver‐reported expressive word count was transformed into age and gender adjusted percentile ranks using the corresponding standard tables in the FRAKIS manual. For group comparisons and longitudinal predictions, caregivers' responsiveness and initiative pointing was combined across paradigms to obtain the best proxy to caregivers' natural behavior. Caregivers' responsiveness was operationalized as a proportion (number of contingent responses/(infant gestures + vocalizations)). Caregivers' initiative pointing frequency was calculated per minute of undisturbed data collection. To investigate a possible cross‐sectional direct link between caregivers' responsiveness, initiative pointing, and infants' gesture use, we focused on caregivers' behavior during the corresponding paradigm; for example the effect of caregivers' responsiveness during the remote decorated room on infants' pointing during the remote decorated room.

Our main analysis assessed whether group assignment influenced infants' communicative development. At t1, only one infant pointed and two infants used HoG gestures, so that these data were not further analyzed. We used independent *t*‐tests to assess the effect of group assignment on infants' HoG and pointing frequency at t2, and their expressive language skills at t3. To obtain an indication of whether parents adapted their behaviors according to group assignment, we compared caregivers' responsiveness and initiative pointing between groups and time points with mixed 2 × 2 ANOVAs (between subject: group, within subject: time (t1, t2)).

We conducted regression analyses to further assess longitudinal and cross‐sectional relations between infants' communicative development and caregivers' behavior while controlling for group assignment. On the longitudinal level, we assessed whether natural variance in caregivers' behaviors at t1 would predict infants' early HoG and pointing use at t2. The two longitudinal models on infants' expressive language development assessed the predictive role of caregivers' behaviors at t1 and t2, respectively. Additionally, we included infants' HoG and pointing frequency at t2 as control variables, because these have previously been found to predict language outcomes.

On the cross‐sectional level, we assessed whether caregivers' behaviors at the outcome assessment at t2 would relate to infants' gesture use in the same setting, as an effect of direct mutual adapting, and whether the group assignment would have an effect beyond cross‐sectional, situational adapting. Infants' age at t2 was included in most regression analyses as a control variable to predict infants' gesture frequencies. As the dependent variable of infants' expressive language abilities was already adjusted for infants' age, we did not include age as an additional a control variable in the corresponding models.

We compared each regression model to a model including only the control variables, or a null‐model in the case of infants' language development, via likelihood ratio test. The amount of explained variance (McFaddens *R* ^2^, Pseudo *R* ^2^) is reported for significant models. Infants' pointing and HoG frequency were predicted with negative binomial regressions for count data (Venables and Ripley 2002), infants' expressive language abilities with beta regression models for proportional outcomes (Cribari‐Neto and Zeileis 2010).

## 6 Results

Descriptive data on infant and caregiver behavior in the training and the control group are presented in Table 1. Forty percent of all infants were index‐finger pointers (Pointer) at 10 months, and infants' pointing frequency ranged from 1 to 41. Forty‐five percent of all infants used HoG gestures during free play (HoGer) at 10 months and the frequency ranged from one to 14. Infants' mean percentile concerning their expressive lexicon was 59 (SD = 27.95). At t1, 11% of all caregiver pointing gestures were responsive (66 out of a total of 584 points) and at t2, 15% of all caregiver pointing gestures were responsive (143 out of a total of 931 points). Caregivers' responsive and initiative pointing correlated longitudinally from t1 to t2, and cross‐sectionally at t1 and t2 (see Table A1).

**TABLE 1:** Descriptive data.

	Control group			Training group		
	t1	t2	t3	t1	t2	t3
Infants						
HoGer	1	12	—	1	24	—
HoG frequency	0.02 (0.07)	0.13 (0.22)	—	0.00 (0.03)	0.33 (0.55)	—
Pointer	0	16	—	1	18	—
Pointing frequency	0.01 (0.07)	0.38 (0.79)	—	0.03 (0.19)	0.40 (1.32)	—
Language	—	—	63 (24)	—	—	56 (30)
Caregivers						
Responsiveness	0.69 (0.26)	0.69 (0.22)	—	0.68 (0.25)	0.79 (0.16)	—
Deco	0.70 (0.25)	0.72 (0.30)		0.70 (0.27)	0.82 (0.22)	
Free play	0.68 (0.30)	0.65 (0.29)		0.64 (0.35)	0.77 (0.23)	
Initiative pointing	0.56 (0.68)	0.83 (0.81)	—	0.56 (0.71)	1.17 (0.98)	—
Deco	1.11 (1.35)	1.41 (1.53)		1.11 (1.44)	2.03 (1.81)	
Free play	0.22 (0.31)	0.27 (32)		0.13 (0.21)	0.30 (0.37)	

*Note:* Behavioral means with standard deviations in parentheses. The data on infants' HoG development was collected during remote free play. The data on infants' pointing development was collected during the remote decorated room. Language = gender and age specific percentile regarding infants' expressive language abilities, *t*1 = 7 months, *t*2 = 10 months, *t*3 = 24 months, Deco = remote decorated room, Free play = remote free play.

### 6.1 Group Comparisons

The following analyses assessed whether infants' gesture use at t2 and their language abilities at t3 differed between the control and the training group. At t2, infants used significantly more HoG gestures in the training group compared to infants in the control group, *t* (54.78) = −2.18, *p* = 0.034. At t2, twice as many infants were HoGers in the training group compared to the control group, *χ* ^2^ (1) = 5.70, *p* = 0.017. The difference in infants' pointing frequency between groups at 10 months was not significant, *t* (80) = ‐0.06, *p* = 0.956. At 24 months infants expressive language abilities did not differ between groups, *t* (62) = 0.76, *p* = 0.452.

The following analyses assessed whether caregivers' behavior differed between the control and the training group at t1 and t2. The 2 (between subject: group) x2 (within subject: time) ANOVA on caregivers' responsiveness revealed no main effect of group, *F* (1,80) = 0.93, *p* = 0.339, a main effect of time point, *F* (1,80) = 4.12, *p* = 0.046, and a significant interaction, *F* (1,80) = 4.13, *p* = 0.045. Follow‐up comparisons indicated that caregivers' responsiveness at t1 did not differ between groups, *F* (1,80) = 0.08, *p* = 0.784. At t2, caregivers in the training group were more responsive than caregivers in the control group, *F* (1,80) = 5.09, *p* = 0.027. While caregivers in the control group were equally responsive at t1 and t2, *F* (1,80) = 0.00, *p* = 0.997, caregivers in the training group increase their responsiveness from t1 to t2, *F* (1,80) = 8.47, *p* = 0.005.

The 2 (between subject: group) x2 (within subject: time) ANOVA on caregivers' initiative pointing frequency revealed no main effect of group, *F* (1,80) = 1.13, *p* = 0.291, a main effect of time, *F* (1,80) = 25.92, *p* < 0.001, and a significant interaction, *F* (1,80) = 4.15, *p* = 0.045. Follow‐up comparisons indicated that caregivers' initiative pointing at t1 did not differ between groups, *F* (1,80) = 0.00, *p* = 0.954. At t2, caregivers in the training group tended to use more initiative pointing gestures compared to caregivers in the control group, albeit not statistically significantly more, *F* (1,80) = 2.92, *p* = 0.092. Caregivers increased their initiative pointing frequency from t1 to t2 in the training group, *F* (1,80) = 26.05, *p* < 0.001, and in the control group, *F* (1,80) = 4.55, *p* = 0.036.

### 6.2 Longitudinal and Cross‐Sectional Relations

Longitudinally, group assignment was predictive of infants' HoG frequency at 10 months, while neither caregivers' responsiveness nor their initiative pointing at 7 months yielded a significant effect, *χ* ^2^ (3) = 7.64, *p* = 0.054, McFaddens *R* ^2^ = 0.32 (see Table A2; A). Infants' pointing frequency at 10 months was neither explained by group assignment, nor by caregivers' responsiveness or their initiative pointing at t1, *χ* ^2^ (3) = 0.30, *p* = 0.961 (see Table A2; B). Infants' expressive language abilities at 24 months were neither explained by group assignment, nor by caregivers' responsiveness or their initiative pointing at t1, *χ*2 (3) = 6.41, *p* = 0.093, or at t2, *χ*2 (3) = 2.79, *p* = 0.426 (see Table A3; A & B). However, infants' expressive language abilities in both models were positively associated with infants' pointing frequency at 10 months explaining respectively 16% and 20% of variance (pseudo *R* ^2^) in infants' language abilities.

Cross‐sectionally, group assignment remained a significant predictor of infants' HoG frequency at 10 months, which was additionally associated with caregivers' responsiveness during free play at t2, *χ* ^2^ (2) = 12.74, *p* = 0.002, McFaddens *R* ^2^ = 0.53 (see Table 2; A). Infants' pointing frequency at 10 months was neither predicted by group assignment, nor by caregivers' responsiveness or initiative pointing frequency in the decorated room at t2, *χ* ^2^ (3) = 2.34, *p* = 0.506 (see Table 2; B).

**TABLE 2:** Cross‐Sectional regression models on infants' gesture development.

		B(SE)	Exp(B)	95% CI, exp(B)		*p*
				LL	UL	
A	Group, 0 = control	0.80 (0.37)	2.23	1.08	4.61	**0.029**
HoG, *N* = 82	Responsiveness, t2, FP	2.33 (0.79)	10.25	2.13	58.13	**0.003**
	Initiative pointing, t2, FP	−0.39 (0.56)	0.67	0.22	2.11	0.479
	Infant age, t2	0.00 (0.02)	1.00	0.96	1.04	0.909
	Intercept	−2.82 (5.83)	0.06	0.00	19,670.48	0.628
		deviance = 71.93; df = 77; *NegBin* = 0.68				
B	Group, 0 = control	−0.08 (0.55)	0.92	0.27	2.97	0.883
Pointing, *N* = 80	Responsiveness, t2, D	1.63 (1.12)	5.08	0.12	207.41	0.146
	Initiative pointing, t2, D	−0.29 (0.18)	0.75	0.52	1.11	*0*.*094*
	Infant age, t2	. 02 (0.03)	1.02	0.94	1.11	0.445
	Intercept	−6.72 (8.68)	0.00	0.00	27,342,803.01	0.439
		deviance = 58.51; df = 75; *NegBin* = 0.19				

*Note:* T1 = 7 months, *t*2 = 10 months, FP = remote free play, *D* = remote decorated room, deviance = residual deviance. The bold values indicate statistically significant *p*‐values (*p* < 0.05).

## 7 Discussion

The current study investigated the influence of caregivers' responsiveness on infants' communicative development in the context of a caregiver training in a *German* longitudinal sample. The main finding was that providing caregivers with educational training advice and examples to enhance their responsivity at 7 months of age promoted infants' early HoG gesture use at 10 months of age. In addition, findings confirmed the well documented relation between infants' pointing and their subsequent language abilities, extending it to the early onset of pointing gestures at 10 months of age. Caregivers' early behaviors recorded at 7 and 10 months of age were not related to infants' early pointing and their later language skills.

The group difference effect on infants' HoG gestures early in development suggests that caregivers' responsiveness plays a crucial role in the early emergence of proximal object‐directed gestures. We cannot know whether the daily 15 min interacting with infants drove the effect irrespective of the type of instructions, because we opted for a naturally developing comparison group without intervention. We find it unlikely, however, that the caregiver groups simply differed in the amount of their daily interactions with their infants. Instead, we assume that the educational intervention changed the quality of interaction. We cannot know exactly how caregivers implemented our conceptual instructions on a daily basis, and concretely by which behaviors, as it is methodologically and analytically not feasible to track any and all behaviors continuously and exhaustively in natural interactions across months. In contrast, our approach targeted caregivers' conceptual understanding of the importance and use of responsiveness. To this end, the instructions were educational and kept rather simple, and were illustrated and exemplified, including videos and clarification questions. Further, we kept in contact with the caregivers monthly and encouraged them to keep track weekly of their implementation, which likely helped reminding them of the intervention and kept them motivated. Inducing a change in the mind‐set of caregivers through our instructions should affect their responsive behaviors. To this end, we measured caregivers' behaviors during the beginning and end of the intervention. While these measures do not reveal what caregivers did during the entire intervention period, the results revealed a clear influence of the instruction on caregivers' behaviors: Caregivers successfully implemented the educational instructions as revealed by increased responsive behaviors after the intervention compared to the control group.

Importantly, our cross‐sectional analyses revealed that the group differences were not exclusively driven by immediate effects of increased caregivers' responsiveness at 10 months during the assessment, as a form of mutual communicative adapting in the immediate situation. Instead, the 3 months earlier group assignment still had an independent effect. Further, the group differences were not just due to a few infants gesturing with high frequency, but apparent on the individual level, with about twice as many infants using HoG gestures in the training group. Together, these findings support the notion that a process of social co‐construction runs deep in the ontogeny of early object‐related communication. The early influence of responsiveness in the emergence of gestures (i.e., from 7 to 10 months) suggests that social responses not only act on these gestures once they have emerged around 10 months; instead, they bring about their very emergence.

The absence of training effects on infants' pointing frequency may be explained by the fact that most infants only begun to point shortly before the 10‐month‐assessment (and more than half of the sample did not point then). One possibility is that the direct training period was too short to unfold promoting effects. This logistic shortcoming possibly also affected the interindividual variance of infants' pointing frequency. While several design features may contribute to explaining the lack of training effects on infants' pointing frequency, it is important to stress that the absence does not meaningfully reject the idea that the training of caregivers' responsiveness influences infants' pointing, albeit perhaps a few months later (Kaletsch and Liszkowski, 2025). The absence of training effects on infants' language development may suggest that the 14 months between the end of the intervention and infants' language assessment are a too long period to reveal stable continuity. If the training would have continued, and caregivers would have continued to display increased responsiveness after the course of the study, one could expect promoting effects on infants' pointing frequency, and consequently also on infants' language abilities. It is noteworthy, however, that infants' early pointing was predictive of their subsequent language development a year later. It supports the idea that caregiver behaviors alone are not the sole driver in language development, which requires in addition a social‐cognitive, communicative competence underlying infants' use of pointing (Liszkowski and Kaletsch, in press).

The natural variance in caregivers' responsiveness at 7 months, that is before training, did not predict infants' HoG and pointing frequency or infants' language abilities. One possibility is that the natural variance in our rather homogeneous sample was not large enough. Another possibility in hindsight is that responsiveness changed over the developmental period with regard to the infants' signals and activities. At 7 months, responses mostly addressed infants' vocalizations, and the infants were often in prone or supine position while surrounded by objects, but not extensively exploring and acting on these. So, caregivers' responsiveness at 7 months may have initially rather supported general communicative practice, like addressing and turn‐taking, than specific object‐directed communication. Across the subsequent 3‐month training period, responsiveness likely changed to incorporate more object exchanges (see Liszkowski and Rüther, 2021). Future studies may assess changes in what caregivers respond to as a function of infants' developing object‐related activities.

Caregivers' initiative pointing frequency was neither longitudinally nor cross‐sectionally related to infants' communicative development. One possibility is that caregivers' initiative pointing enhances object‐directed gestures slightly later, around 10–12 months, as a training study by Rowe and Leech, (2018) suggests (but see Matthews et al. 2012). Further, we conceptually distinguished caregivers' responsive pointing gestures (which were included in the responsiveness construct) from initiative gestures which do not respond to infants' communicative signals. Tomasello and Farrar (1986) argued that caregiver gestures which direct attention away from infants' current focus, instead of following into their focus, complicate the establishment of joint attention, yielding a negative relation to infants' vocabulary development (Garner and Landry 1994; Legerstee et al. 2002). In the current study, in the context of the remote deco room, only a few points were responsive in total. However, the rest of the points, coded inversely as initiative points, were mostly not points that directed attention away to something completely different, but rather points meant to maintain attention to the display screen. The current setting did not distinguish well between a general shared focus on the screen and details displayed on the screen. Other settings might be more suited to isolate ‘direct‐away’ points from temporally contingent points and from thematically contingent points, to investigate influences of these different qualities of caregiver pointing.

Lean imitation accounts on the development of infants' pointing, according to which infants simply mimic caregivers' pointing gestures, are not well supported by the findings of this study. However, infants may still learn about pointing by observing caregivers' pointing in responsive contexts. In this respect, it is noteworthy that caregivers increase their pointing from 7 to 10 months (see Rüther and Liszkowski, 2023 for a similar finding), which suggests that caregivers naturally adjust their use of pointing to infants' communicative development, that is, caregivers increase their pointing when infants become ready to process and learn from it, before they point themselves.

The current study supports the proposition that increased responsiveness early in ontogeny enhances infants' object‐directed communication early on, specifically the emergence of HoG gestures. While some accounts emphasize the socialization of gesture after its emergence, from a non‐communicative to a communicative use (Carpendale and Carpendale, 2010), in our view the communicative and interactional activities of both interactant partners bring the gesture into existence (Liszkowski and Rüther, 2021). On the latter account, caregiver responsiveness should impact the early emergence of HoG gestures, as corroborated by the findings of the current study. From that perspective, infant deictic gestures are communicative as they emerge. A further finding of the current study supports this proposition: the early emerged pointing gestures at 10 months were already predictive of later language acquisition, suggesting that they are communicative from the beginning.

Our interpretation aligns with co‐construction and scaffolding theories (Bruner, 1983; Vygotsky and Cole, 1978), which hold that caregivers adapt their responses to infants' communicative skills. On this view, it is not simply any communicative input which shapes infants' behavior into communication. Instead, effective responsiveness depends on infants' interactional skills, as they need to process these responses meaningfully. This also suggests that enhancing caregiver responsiveness through interventions should be more effective on a conceptual‐educational level, as in the current study, than targeting any specific caregiver behavior in isolation. This may be especially relevant to applied settings targeting longer lasting effects across development, during which infants' skills and behaviors, and accordingly caregivers' responsive behaviors change. Current findings suggest that the dialectic developmental spiral, which encompasses infants' communication and caregivers' responses, runs deep in ontogeny, yielding incipient early forms of object‐directed communication, and may be used by intervention programs already in the first year of life.

## Author Contributions

**Katharina Kaletsch:** conceptualization, investigation, writing – original draft, methodology, visualization, writing – review and editing, software, formal analysis, project administration, data curation. **Ulf Liszkowski:** conceptualization, writing – review and editing, supervision, resources, validation, methodology, formal analysis, project administration, writing – original draft.

## Funding

The authors have nothing to report.

## Ethics Statement

The present study was conducted according to guidelines laid down in the Declaration of Helsinki, with informed consent obtained from a caregiver for each child before any assessment or data collection. All procedures in this study were approved by the local ethics committee at the *University of Hamburg*.

## Conflicts of Interest

The authors declare no conflicts of interest.

## Data Availability

The data that support the findings of this study are available from the corresponding author [Katharina Kaletsch] upon reasonable request. A preprint is available [https://doi.org/10.31234/osf.io/5jxap_v1] including training materials in English and German [https://osf.io/sx84w/files/osfstorage].

## Appendix

**TABLE A1:** Correlations between caregivers' responsiveness and initiative pointing.

		1	2	3
1. Responsiveness, t1	r *p* N			
2. Responsiveness, t2	r *p* N	0.35 **0.001** 82		
3. Initiative pointing, t1	r *p* N	0.30 **0.006** 84	0.21 *0*.*059* 82	
4. Initiative pointing, t2	r *p* N	0.31 **0.005** 82	0.28 **0.010** 82	54 **< 0.001** 81

*Note:* Caregivers' responsiveness and initiative pointing were combined across paradigms (remote decorated room + remote free play). The bold values indicate statistically significant *p*‐values (*p* < 0.05).

**TABLE A2:** Longitudinal regression models on infants' gesture development.

		95% CI, Exp(B)				
		B(SE)	Exp(B)	LL	UL	*p*
A	Group, 0 = control	0.91 (0.37)	2.49	1.20	5.23	**0.015**
HoG, *N* = 82	Responsiveness, t1	0.63 (0.78)	1.88	0.45	7.57	0.416
	Initiative pointing, t1	−0.40 (0.31)	0.67	0.35	1.28	0.197
	Infant age, t2	0.01 (0.02)	1.01	0.97	1.06	0.516
	Intercept	−4.52 (5.96)	0.01	0.00	6369.55	0.448
		Deviance = 73.31; df = 77; *NegBin* = 0.57					
B	Group, 0 = control	0.12 (0.55)	1.11	0.37	3.32	0.848
Pointing, *N* = 81	Responsiveness, t1	0.11 (1.17)	1.11	0.13	7.49	0.927
	Initiative pointing, t1	0.24 (0.43)	1.27	0.48	4.55	0.575
	Infant age, t2	0.04 (0.03)	1.04	0.96	1.12	0.169
	Intercept	−11.97 (8.99)	0.00	0.00	187,742.27	0.183
		Deviance = 58.53; df = 76; *NegBin* = 0.18				

*Note:* Caregivers' responsiveness and initiative pointing were combined across paradigms (remote decorated room + remote free play). The bold values indicate statistically significant *p*‐values (*p* < 0.05).

**TABLE A3:** Longitudinal regression models on infants’ language development.

		B (SE)	95% CI, (B)		*p*
			LL	UL	
A	Group, 0 = control	−0.22 (0.25)	−0.71	0.28	0.390
Expressive language, *N* = 64	Infant pointing, t2	0.45 (0.12)	0.22	0.68	**< 0.001**
	Infant HoG, t2	−0.24 (0.27)	−0.77	0.01	0.379
	Responsiveness, t1	−0.38 (0.49)	−1.33	0.57	0.435
	Directive pointing, t1	−0.37 (0.19)	−0.75	0.01	*0*.*056*
	Intercept	0.83 (0.36)	0.06	1.38	0.020
B	Group, 0 = control	−0.24 (0.26)	−0.76	0.27	0.355
Expressive language, *N* = 64	Infant pointing, t2	0.36 (0.12)	0.13	0.60	**0.003**
	Infant HoG, t2	−0.38 (0.30)	−0.97	0.20	0.199
	Responsiveness, t2	0.98 (0.73)	−0.44	2.40	0.177
	Directive pointing, t2	−0.19 (0.15)	−0.49	0.11	0.224
	Intercept	−0.07 (0.46)	−0.98	0.84	0.876

*Note:* Caregivers' responsiveness and initiative pointing were combined across paradigms (remote decorated room + remote free play). The bold values indicate statistically significant *p*‐values (*p* < 0.05).
